# Encapsulated papillary carcinoma of the breast: An institutional case series and literature review

**DOI:** 10.1002/cam4.5855

**Published:** 2023-03-31

**Authors:** Hiang Jin Tan, Puay Hoon Tan, Lester Chee Hao Leong, Veronique Kiak Mien Tan, Benita Kiat Tee Tan, Sue Zann Lim, Madhukumar Preetha, Chow Yin Wong, Wei Sean Yong, Yirong Sim

**Affiliations:** ^1^ SingHealth Duke‐NUS Breast Centre Singapore Singapore; ^2^ Department of Breast Surgery Singapore General Hospital Singapore Singapore; ^3^ Division of Pathology Singapore General Hospital Singapore Singapore; ^4^ Duke‐NUS Medical School Singapore Singapore; ^5^ Department of Diagnostic Radiology Singapore General Hospital Singapore Singapore; ^6^ Division of Surgery and Surgical Oncology National Cancer Centre Singapore Singapore Singapore; ^7^ Department of General Surgery (Breast service) Sengkang General Hospital Singapore Singapore

**Keywords:** breast, encapsulated, papillary

## Abstract

**Background:**

Encapsulated papillary carcinoma of the breast is rare, making difficult diagnosis and resulting in patients undergoing excision biopsy before definitive surgery. Evidence‐based guidelines are sparse. We would like to further elucidate the clinicopathological, treatment and survival outcomes.

**Materials and Methods:**

54 patients identified, with a median follow up duration of 48 months. Patients' demographics, radiological and clinicopathological characteristics, treatment, adjuvant therapies as well as survival data were analysed.

**Results:**

18 (33.3%) cases were pure EPC, 12 (22.2%) were EPC associated with ductal carcinoma in situ (DCIS) and 24 (44.4%) cases had concurrent invasive ductal carcinoma. EPCs were more likely to present as a solid‐cystic mass on sonography (63.8%), regular‐shaped (oval or round) (97.9%), lack spiculations (95.7%) and lack suspicious microcalcifications (95.6%). Median tumour size was largest in the EPC with IDC group (18.5 mm). 2 patients developed loco‐regional recurrence. Overall survival is good for EPCs of all subtypes.

**Conclusion:**

EPC is a rare tumour with excellent prognosis.

## Graphical abstract

Encapsulated Papillary Carcinoma (EPC) is a rare tumour mainly affecting post‐menopausal woman with excellent prognosis, with no significant differences in overall survival between all three groups ‐ pure EPC, EPC with DCIS and EPC with IDC. The presence of a solid component within a cystic lesion of the breast should raise the clinician's suspicion of malignancy, and due care has to be taken when doing a core biopsy, to ensure samples are obtained from the solid component of these lesions.

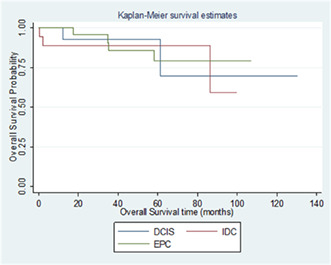

## 1 INTRODUCTION

Encapsulated papillary carcinoma of the breast (EPC) is a rare type of papillary neoplasm. It accounts for 0.5–1% of all breast cancers.^1^ Described as a well‐ circumscribed malignant papillary mass surrounded by thick fibrous capsule, EPCs typically presents in postmenopausal woman (between 55 to 67 years of age).^2^, ^3^ It is generally considered as a low risk cancer, with a 5% local recurrence risk and an overall favourable prognosis.^4^ Although it is debatable if EPCs should be treated as an in‐situ or invasive carcinoma,^4^ EPCs are often managed as an in‐situ disease because of its indolent clinical behaviour. EPCs uniquely express a pattern of invasion‐ associated markers between DCIS and invasive breast cancer (IBC). On one hand, EPC is notoriously difficult to diagnose pre‐operatively with needle biopsy due to its solid cystic nature. On the other hand, it can mimic an invasive carcinoma if there is presence of entrapped breast epithelium along the needle tract after core biopsy, causing confusion to the clinician. There have also been several reported cases of its association with invasive cancer and metastasis.^4^, ^5^, ^6^, ^7^ Besides the few reported series in the literature, there is still a sparsity of data regarding clinicopathologic features of EPC.^8^ There is also an ongoing debate regarding whether EPC is in situ of invasive carcinoma, with no clear agreement among different studies.^4^ Although rare, there are reported cases of high grade EPC, with aggresive histological features and clinical behaviour.^9^ EPCs can be classified as either an invasive or non‐invasive EPC, with the latter further subdivided into a diffuse or a localised form.^6^ More commonly, EPCs can be classified into three main groups: EPC alone (pure form EPC), EPC with ductal carcinoma in situ (DCIS), and EPC with invasive carcinoma.^10^, ^11^ The aim of our study is to retrospectively study, and correlate the characteristics and outcomes of EPCs in our local institution. To date, this is one of the largest series available in the literature.

## 2 METHODS

A retrospective review of our Joint Breast Cancer Registry (JBCR), a prospective database, identified a total of 12,028 patients diagnosed with breast cancer in the Singapore General Hospital and the National Cancer Centre Singapore from January 2004 to December 2017. Inclusion criteria were age > 21 and < 90 years old, patients who were diagnosed with EPC and underwent breast surgery, in the absence of distant metastasis. Patients who declined surgery were excluded. There were 10,427 invasive breast cancers, 1601 ductal carcinoma in‐situ (DCIS) and only 54 patients with EPC. EPC was diagnosed based on criteria described in the WHO classification of tumours of the breast by dedicated breast pathologists.^10^ Clinical variables such as demographics data, clinical, radiological and pathological characteristics, treatment as well as survival data were analysed. Survival time was defined as time from date of diagnosis to date of death or date last seen. The study was reviewed and approved by the Singhealth Institutional Review Board. (CRIB Ref No: 2019/2419).

### 2.1 Statistical analysis

Patients' demographics, radiological and clinicopathological characteristics, treatment, adjuvant therapies as well as survival data were analysed. Patients' baseline characteristics are expressed as median and mean for continuous data and as numbers with percentages for categorical data, as appropriate. The normality of continuous variables was examined, and all between‐group differences of non‐normally distributed continuous variables were tested using nonparametric statistics. Cumulative overall survival and disease‐free survival rates were determined using the Kaplan–Meier method. All statistical analysis was performed using commercially available software (SPSS, version 26.0, for Windows; SPSS, Inc).

## 3 RESULTS

The patients' basic demographic data are summarised in Table 1. A total of 54 patients were included in this study. Among the 54 cases, 18 cases were pure EPCs (33.3%), 12 (22.2%) were EPCs associated with ductal carcinoma in‐situ (DCIS) and 24 (44.4%) cases had concurrent invasive ductal carcinoma (IDC). The majority of patients were ethnically Chinese (50 patients, 92.6%), a reflection of the Chinese majority in our population.

**TABLE 1:** Patient's demographic data of encapsulated papillary carcinoma (EPC) of breast.

Patient demographics	Pure EPC *n* = 18 (%)	EPC with DCIS *n* = 12 (%)	EPC with IDC *n* = 24 (%)
Race			
Chinese	16 (88.9)	12 (100.0)	20 (83.3)
Malay	2 (11.1)	0 (0.0)	3 (12.5)
Indian	0 (0.0)	0 (0.0)	1 (4.2)
Others	0 (0.0)	0 (0.0)	0 (0.0)
Age, median (range)	63.5 (42–83)	59 (49–77)	69.5 (39–87)

Abbreviations: DCIS, ductal carcinoma in situ; IDC, invasive ductal carcinoma.

Table 2 summarises the clinical features and tumour characteristics of the EPC tumours. Student's *T* test was conducted to perform the statistical analysis. The median size of tumour was largest in the EPC with IDC group, 18.5 mm (range 7–78 mm), followed by pure EPC group, 19.5 mm (range 2.5–50 mm) and lastly the EPC with DCIS group, with a median size of tumour at 15.5 mm (range 5–45 mm). The majority of the patients (*n* = 36, 66.7%) presented with symptoms, either of breast lumps (34 patients, 63.0%) or nipple discharge (2 patients, 3.4%). The next most common presentation was the identification of new BIRADS 3 breast nodules on follow up breast surveillance for benign nodules. (12 patients, 22.2%). For these 12 patients, the new nodules were further evaluated and they were diagnosed with EPC.

**TABLE 2:** Clinical features and tumour characteristics of encapsulated papillary carcinoma (EPC) of breast.

	Pure EPC *n* = 18 (%)	EPC with DCIS *n* = 12 (%)	EPC with IDC *n* = 24 (%)
Presentation			
Symptomatic			
Lump	11 (61.1)	7 (58.3)	17 (70.9)
Nipple discharge	0 (0.0)	0 (0.0)	2 (8.3)
Asymptomatic			
Incidental detection on follow up imaging for other breast lesions	4 (22.2)	2 (16.7)	2 (8.3)
Routine screening	3 (16.7)	3 (25.0)	3 (12.5)
Family history of breast cancer	1 (5.6)	2 (16.7)	5 (20.8)
Methods of diagnosis			
Core biopsy	15 (83.3)	6 (50.0)	19 (79.2)
Excision biopsy	3 (16.7)	6 (50.0)	5 (20.8)
Tumour characteristics			
Size, median, mm (range)	17.5 (2.5–45)	15.5 (5–45)	18.5 (7–78)
Grade			
1	4 (22.2)	2 (16.7)	8 (33.3)
2	10 (55.6)	9 (75.0)	15 (62.5)
3	0 (0.0)	0 (0.0)	0 (0.0)
Unknown	4 (22.2)	1 (8.3)	1 (4.2)
Size of IDC, median, mm (range)	NA	NA	6 (0.5–30)
Nodal metastasis	0 (0.0)	0 (0.0)	2 (8.3)
Receptor status			
ER			
Positive	15 (83.3)	10 (83.4)	20 (83.3)
Negative	2 (11.1)	1 (8.3)	4 (16.7)
Unknown	1 (5.6)	1 (8.3)	0 (0.0)
PR			
Positive	15 (83.3)	10 (83.4)	20 (83.3)
Negative	2 (11.1)	1 (8.3)	4 (16.7)
Unknown	1 (5.62)	1 (8.3)	0 (0.0)
HER 2			
Positive	NA	NA	4 (16.7)
Negative	NA	NA	20 (83.3)
Unknown	NA	NA	0 (0.0)
LVI + ve	0 (0.0)	0 (0.0)	1 (4.2)

Abbreviations: DCIS, ductal carcinoma in situ; ER, oestrogen receptor; HER2, human epidermal growth factor receptor‐2; IDC, invasive ductal carcinoma; LVI, lymphovascular invasion; MMG, mammogram; PR, Progesterone receptor; US, ultrasound.

Of those patients with EPCs and IDC (*n* = 24), 17 (70.9%) presented with a palpable breast lump, and 3 (12.5%) were detected by mammographic breast screening. 2 (8.3%) patients presented with nipple discharge while the remaining 2 patients were under detected on follow up imaging for benign nodules. There were more patients with family history of breast cancer in the EPC with IDC group (5 patients, 20.8%).

The majority of patients had core biopsy for diagnosis pre‐operatively. Thirteen patients underwent excision biopsy for histological diagnosis, before their definitive surgery. This is because 8 patients had benign appearing radiological features whereas the remaining 5 had prior inconclusive core biopsies, i.e. papillary lesions. EPCs were mostly of low nuclear grade. Majority of the patients were oestrogen and progesterone receptor (ER/PR) positive in all groups. There was no correlation between nuclear grade and the immunohistochemical subtype of the invasive cancers. (Table 3) One out of the three with triple negative invasive cancers had apocrine differentiation. There is no significant difference in the grades of EPC across all three subtypes (pure EPC vs EPC with DCIS vs EPC with IDC) nor between between the EPC grades across the different invasive breast cancer subtypes. Nonetheless, of those with TNBC invasive cancers, the EPCs are of intermediate grade, and none are of low grade. This may demonstrate the continuum of disease progression towards a more aggressive subtype of breast cancer (TNBC), but the numbers are too small (*n* = 4) to be of any significance and to draw any conclusions. All our invasive tumours are of an early stage—almost all were stage I, and three were stage IIs which are detailed in the Table 3.

**TABLE 3:** Comparision of nuclear grade of EPC and IDC in those EPCs with invasive component, according to their immunohistochemical subtype.

IDC IHC	*n*	EPC grade (*n*)	IDC grade (*n*)	Overall Stage
ER+ve, Her2−	13	Low: 4 Intermediate: 7 High: 0 Unknown: 2	1: 4 2: 5 3: 0 Unknown: 4	All T1N0m0 1 T2N0M0 (IDC Grade 2) 1 T2N1M0 (IDC Grade 2)
TNBC	4	Low: 0 Intermediate: 4 High: 0 Unknown: 0	1: 1 2: 1 3: 1 Unknown: 1	All T1N0M0 1 T1N1M0 (IDC grade 2)
ER+ve, Her 2+	2	Low: 1 Intermediate: 1 High: 0 Unknown: 0	1: 0 2: 1 3: 0 Unknown: 1	All T1N0M0

Abbreviations: ER, oestrogen receptor; IDC, Invasive ductal carcinoma; IHC, immunohistochemistry; PR, Progesterone receptor, TNBC, triple negative breast cancer.

Table 4 demonstrates the correlation between radiological features and invasive disease.

**TABLE 4:** Correlation between radiological features and presence of invasive disease.

Radiological features		Presence of invasive disease (%)	Non‐invasive disease (%)	Total (%)	*p*‐value
Internal Echoes on Sonography Not available: 8	Solid‐cystic	12 (40.0)	18 (60.0)	30 (63.8)	0.916
	Solid	5 (38.5)	8 (61.5)	13 (27.7)	
	Predominantly Solid with Small Cystic Spaces	2 (50.0)	2 (50.0)	4 (8.5)	
Shape Not available: 8	Oval	17 (37.8)	28 (62.2)	45 (95.7)	0.321
	Round	1 (100)	0 (0)	1 (2.1)	
	Irregular	0 (0)	1 (100.0)	1 (2.1)	
Margins Not available: 8	Ill‐defined	17 (63.0	10 (37.0)	27 (57.4)	**<0.001**
	Well‐defined	1 (5.0)	19 (95.0)	20 (42.6)	
Spiculations Not available: 8	Present	1 (50.0)	1 (50.0)	2 (4.2)	1.000
	Not Present	17 (37.8)	28 (62.2)	45 (95.7)	
Suspicious Microcalcifications on Mammography Not available: 10	Present	1 (50.0)	1 (50.0)	2 (4.4)	1.000
	Not Present	15 (34.9)	28 (65.1)	43 (95.6)	

*Note*: Bold indicates statistically significance *p* < 0.001.

The tumours were categorised as BIRADS 3 in 4.0% of the cases with a valid radiological report (2/50) and BIRADS 4 or 5 in 96.0% (48/50). EPCs were more likely to present as a solid‐cystic mass on sonography (63.8%), appear regular‐shaped (oval or round) (97.9%), lack spiculations (95.7%) and lack suspicious microcalcifications (95.6%). Ill‐defined margins were associated with invasive papillary type disease or concomitant non‐papillary invasive tumours (p < 0.001). (Table 4).

Table 5 demonstrates the treatment and survival data of patients with EPCs. In terms of adjuvant treatment, only 2 patients (11.1%) received chemotherapy as they had EPC with IDC with nodal involvement (N1). Radiotherapy was given to 19 patients, instead of the recommended 23 patients. This non‐compliance to radiation therapy for the 4 patients was due to their fear of side effects. The uptake of hormonal therapy was also lower than expected, with 45 patients (83.3%) being ER positive and 45 patients (83.3%) being PR positive but only 31 patients (72.1%) received hormonal therapy. 10 patients from the ER/PR positive group did not receive hormonal therapy as they had a mastectomy for their DCIS, while the remaining 4 patients rejected the treatment, fearing the side effects.

**TABLE 5:** Treatment and outcome of encysted papillary carcinoma (EPC) of breast.

	Pure EPC *n* = 18 (%)	EPC with DCIS *n* = 12 (%)	EPC with IDC *n* = 24 (%)
Treatment			
Surgery			
BCS	8 (44.4)	5 (41.7)	2 (8.3)
BCS + SLNB	0 (0)	0 (0)	6 (25.0)
SM + SLNB	950.0)	7 (58.3)	14 (58.4)
SM + SLNC+AC	1 (5.6)	0 (0)	2 (8.3)
Chemotherapy	0 (0.0)	0 (0.0)	2 (8.3)
Radiotherapy	8 (42.1)	4 (30.8)	7 (29.2)
Hormonal therapy	9 (47.4)	6 (46.2)	16 (66.7)
Outcome			
Recurrence			
Local	2 (10.5)	0 (0.0)	0 (0.0)
Distant	0 (0.0)	0 (0.0)	0 (0.0)
Mortality			
Breast cancer related mortality	0 (0.0)	0 (0.0)	0 (0.0)
Other cause mortality	1 (5.2)	3 (23.1)	4 (16.7)
OS, median (range)	93 (17–131)	57.0 (12–73)	54 (1–241)

Abbreviations: AC, axillary clearance; BCS, breast conserving surgery; DCIS, Ductal carcinoma in situ; IDC, Invasive ductal carcinoma; OS, Overall survival; SLNB, sentinel lymph node biopsy; SM, simple mastectomy.

**FIGURE 1:**
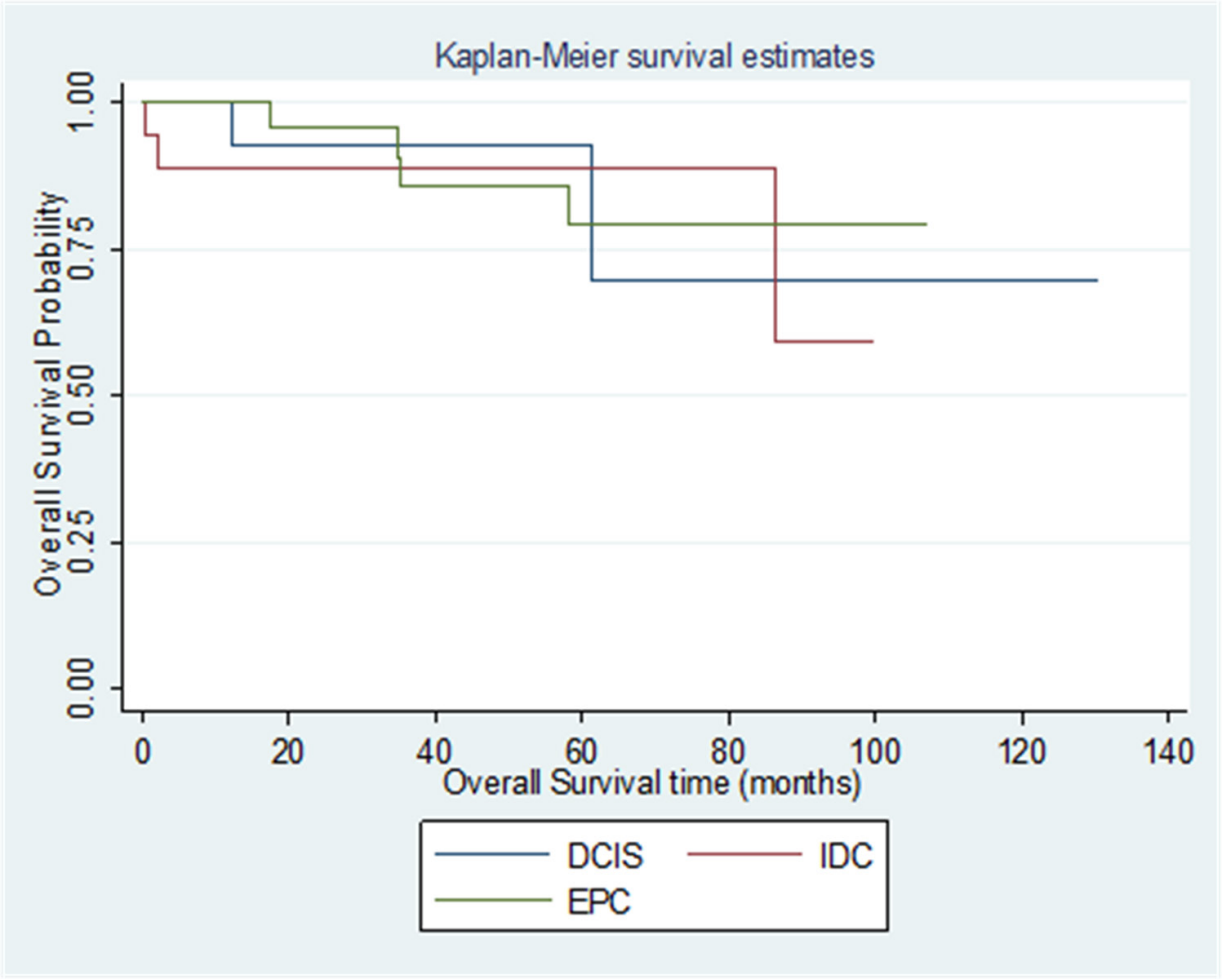
Kaplen Meier curve showing overall survival for all 3 groups – Pure EPC, EPC with DCIS and EPC with IDC.

Median follow up duration was 58.5 months across all 3 subgroups (range:1‐241 months). To date, 2 patients developed loco‐regional recurrence and none had distant metastasis during follow up. Both these patients had pure EPCs, underwent a BCS but declined adjuvant radiotherapy. They developed a recurrence at 36 and 100 months from their initial diagnosis respectively. Nine non‐breast cancer related deaths (15.5%) were reported during follow up: 3 were due to heart disease (despite no radiotherapy to their left breasts), 2 due to pancreato‐biliary cancers, and 4 of unknown causes. Kaplan Meier curve analysis showed no statistical differences for overall survival between all 3 groups. (Figure 1).

## 4 DISCUSSION

EPC is a rare tumour of the breast with a reported incidence of 0.5%–1% in the literature. Our patients are similar to those in reported series, with a median age of 65 years old.^1^, ^3^ There are very few cases reported in the literature of EPCs occurring in women younger than 40 years old.^11^, ^12^ Our series reports a large number of patients, despite its rarity, and demonstrate the difficulty in diagnosing EPC on pre‐operative imaging and biopsies. To our knowledge, our series is the largest series in Asian population. EPCs usually manifest as a painless lump in the breast that could be indolent for several years prior to diagnosis.^3^, ^13^ Our results concur with the findings as the majority of our patients with EPC presented with a palpable breast lump. Similar to the reported literature, the median size of EPCs in this study was 13.5 mm (vs. 20 mm).^4^, ^7^ Patients with EPC associated with IDC reported the highest median size of 25 mm. This is congruent with the literature. EPC can present either as a palpable mass in an otherwise normal breast or as a enlarged breast because of the huge cystic mass within it.^14^ Another commonly reported symptom is that of a bloody nipple discharge.^15^ In our study, the presence of nipple discharge prompted us to proceed to biopsy for 2 patients with otherwise unremarkable BIRADS 3 nodules.

**FIGURE 2:**
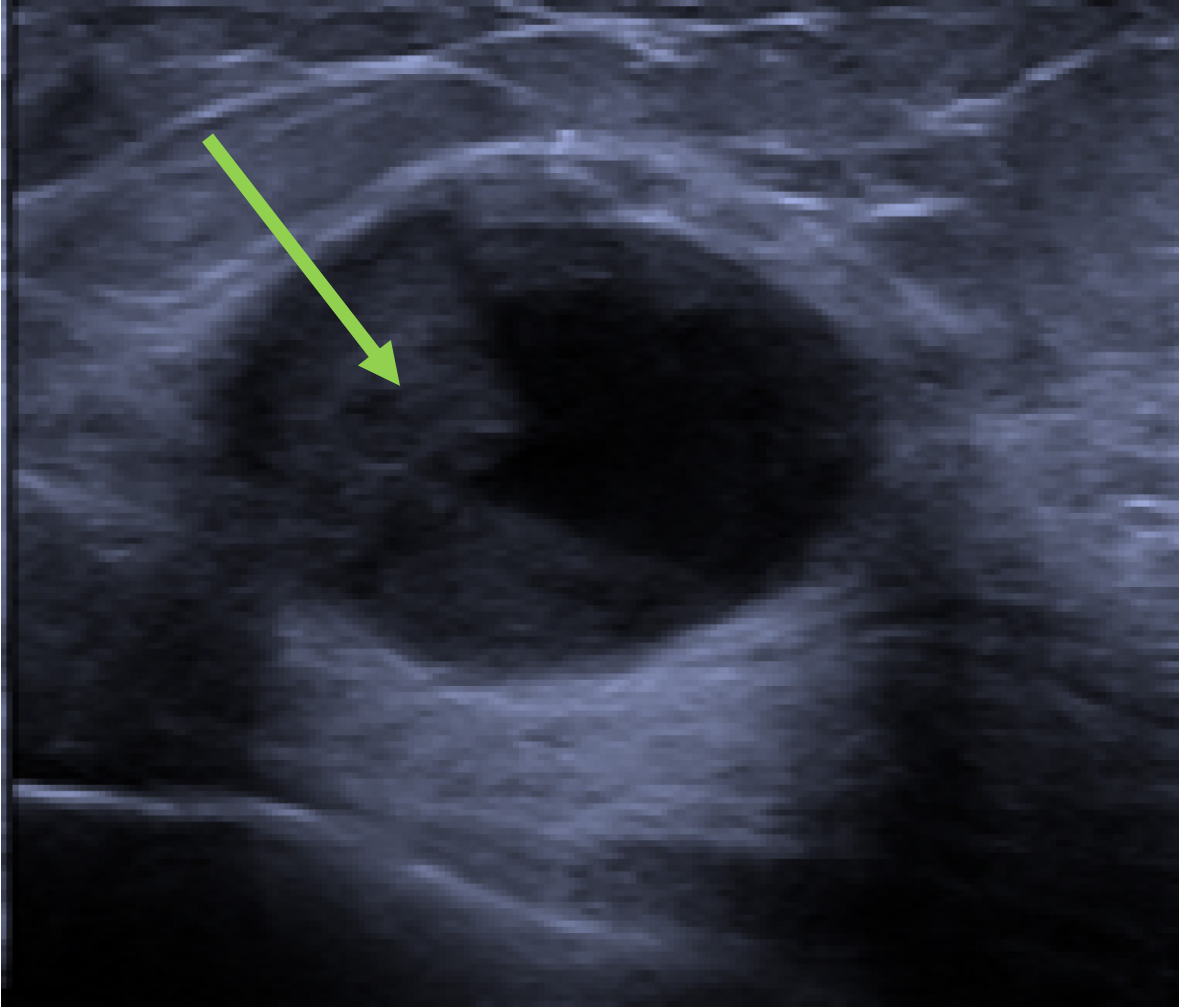
Ultrasound image showing a complex solid‐cystic mass containing an eccentric mural solid component. The margins are partly ill‐defined.

A variety of radiological findings of EPC have been well documented in the literature. It usually presents as a regular shaped or lobulated mass with circumscribed margins near the nipple which lacks spiculations on imaging. Calcifications may not be present. These features were seen in our patients too. The absence of distinctive malignant features predisposes to radiological under diagnosis.^12^ In our series, most of the EPCs presented as a complex cystic mass with a hypervascular intracystic solid component on sonography which should raise the possibility of EPC.^16^ (Figure 2) Ill‐defined margins are suspicious for invasion (*p* < 0.001). Magnetic resonance imaging(MRI) of the breast with contrast enhancement can aid in diagnosis. MRI will revealnhanced cyst wall, septation, and mural nodules.^17^ None of our patients had an MRI of the breast as part of their investigations.

**FIGURE 3:**
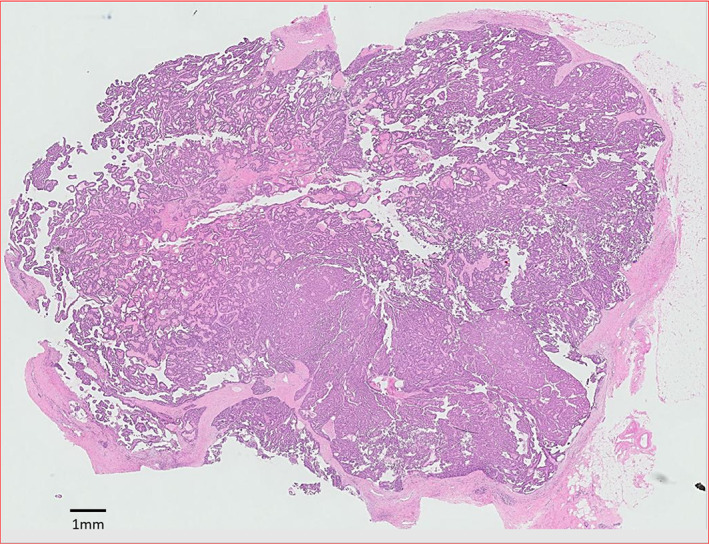
Pure EPC that shows a thick fibrous capsule around coalescent and anastomosing papillary fronds.

**FIGURE 4:**
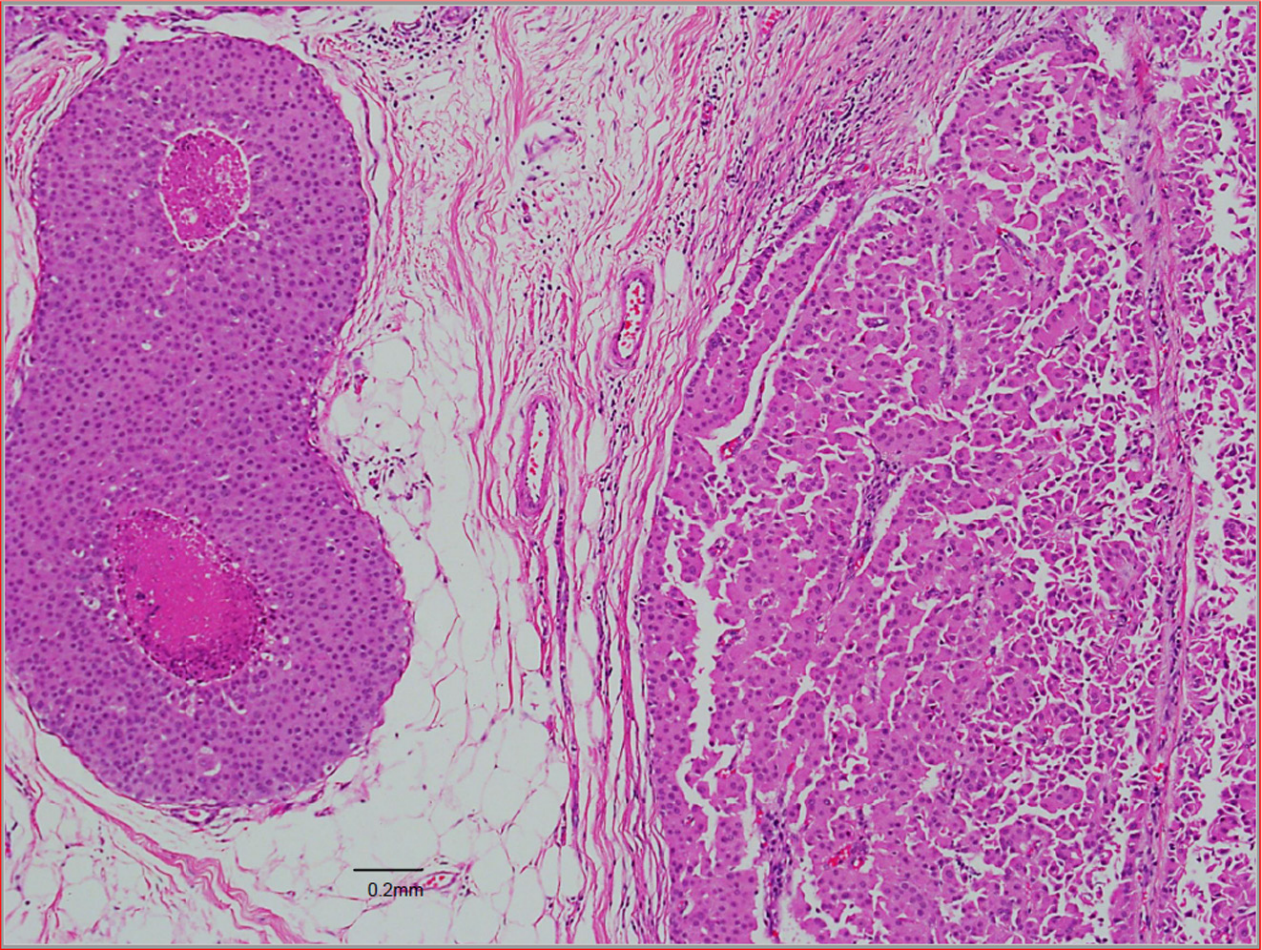
EPC (right field) with DCIS (left field). DCIS showed a solid pattern with necrosis, low to intermediate nuclear grade, adjacent to an encapsulated papillary carcinoma. Both DCIS and encapsulated papillary carcinoma cells disclosed relatively ample pink cytoplasm reminiscent of apocrine morphology.

**FIGURE 5:**
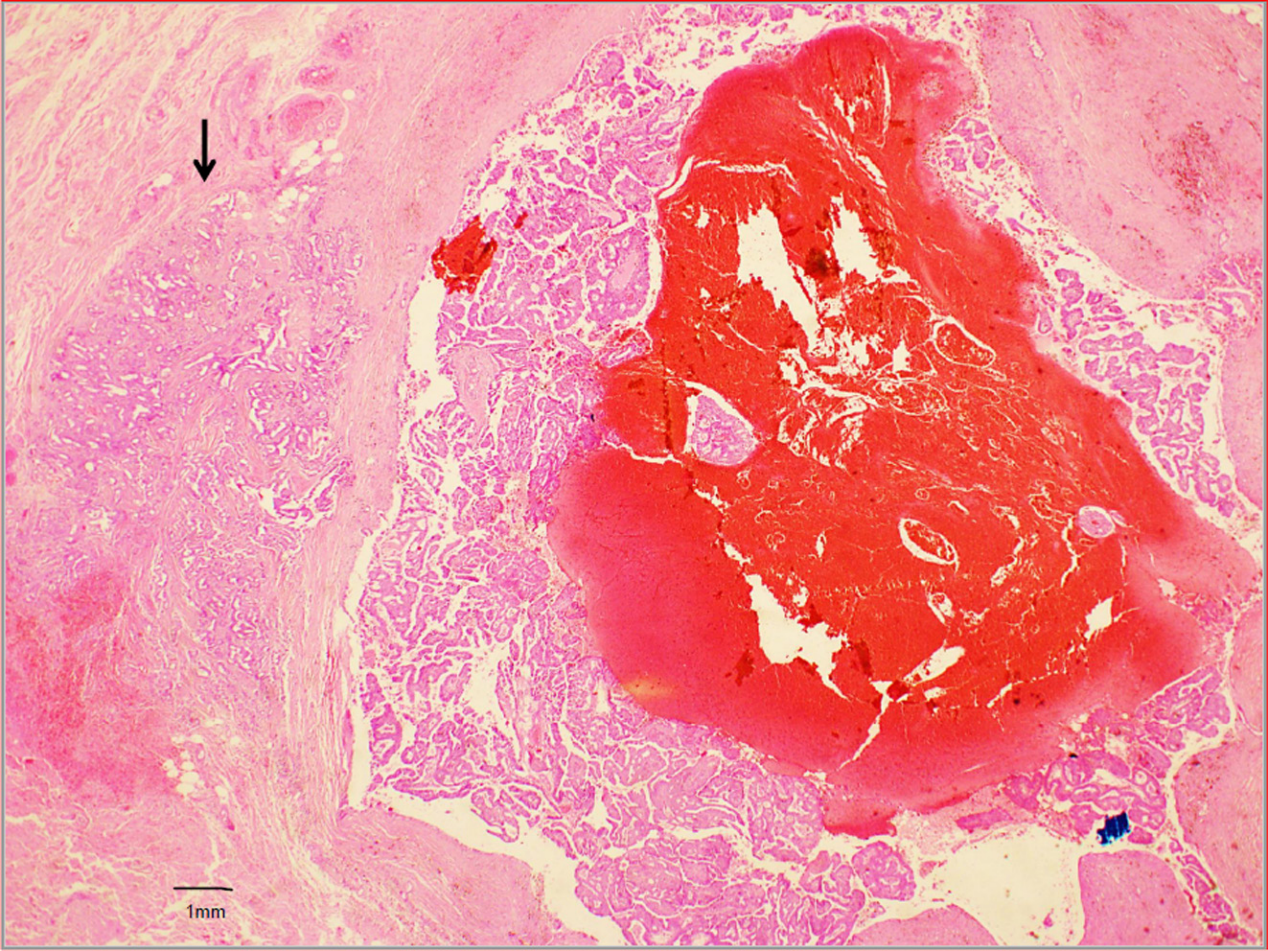
Encapsulated papillary carcinoma shows a fibrous wall and an area of haemorrhage within the tumour. To the left of the encapsulated papillary carcinoma is an area of invasive carcinoma (arrow), where there are crowded tubules within a fibrous stroma.

Core biopsy is the preferred biopsy method and this was done for 43 patients, with definitive diagnoses made in the majority. Only 3 patients (5.2%) received a core biopsy report of a papillary lesion which was inconclusive for malignancy and required excision biopsy to obtain the final diagnosis. 13 patients (22.4%) underwent excision biopsy prior to definitive surgery due to a benign looking lump detected clinically and radiologically. Invasion is usually found in the periphery of the tumour, instead of in the central portion, purportedly due to presence of cystic fluid in the centre of the lesion.^18^ Therefore, extra care is needed by the clinician to biopsy the solid portion of the solid‐cystic lesion to minimise the chance of a false negative sampling. The variety in biopsy techniques may potentially be a confounding factor in diagnosis. Histologically, pure EPC usually demonstrates a thick fibrous capsule around coalescent and anastomosing papillary fronds. (Figure 3) EPC with DCIS will show the additional features of DCIS such as solid, cribriform, micropapillary patterns, with or without necrosis and calcifications, adjacent to the encapsulated papillary carcinoma. (Figure 4) EPC with invasion shows irregular tubules, trabeculae and nests of malignant epithelial within desmoplastic stroma, beyond the fibrous capsule of the EPC. (Figure 5).

The current consensus of the WHO working Group is that pure EPC should be staged and managed as ductal carcinoma in situ.^19^ Management of pure EPC should follow treatment recommendations for DCIS. Surgical excision with negative surgical margins with or without hormonal therapy should be adequate for pure EPC. The role of axillary surgery remains debatable. Some authors argue against axillary lymph node surgery due to the low metastatic rate of this tumour and the morbidity associated with axillary lymph node clearance while there have been reported cases of lymph node metastasis.^1^, ^20^ In our series, 35 patients had lymph node evaluation as they underwent mastectomy with routine concurrent sentinel lymph node biopsy as per protocol. Of these, only those with an associated component of IDC had nodal metastasis in their sentinel node (2 patients). The large proportion of mastectomies in our study may be due to smaller breast volumes, and hence larger tumour to breast ratios in our local Asian population. In addition, cultural preferences may also contribute to the majority of our cancer patients (in general) preferring mastectomies over breast conserving surgery.

Many centres advocate the use of adjuvant radiotherapy and hormonal therapy, but their role in prognosis improvement in pure EPC cases is inconclusive.^5^, ^6^, ^21^ Adjuvant therapy with radiotherapy is recommended if the tumour is associated with DCIS or invasive component in patients who opted for breast conserving surgery or in patients with N2 disease.^1^ In a series of 40 patients with pure EPC, EPC associated with DCIS and EPC associated with IDC, Solorzano et al^21^ reported that the use of radiation did not influence recurrence or survival. Conversely, in a similar study, Fayunju et al^1^ found that patients with EPC associated with DCIS or IDC more frequently undergo adjuvant radiotherapy compared to patients with pure EPC but did not report on the difference in outcomes. In our study, only 16 of 23 patients with breast conserving surgery (64%) underwent adjuvant radiotherapy. This is because some of our patients refused radiotherapy, fearing its side effects.

Hormonal therapy should be advised for patients with positive receptor status and has invasive disease. The use of tamoxifen / letrozole is common when EPC is associated with ER/ PR positive status.^22^ In our series, majority was ER/ PR positive (83.3% and 83.3% respectively) and only 4 patients (7.4%) were HER2 positive. This is consistent with what was reported in the literature.^1^ However, not all of the patients with hormonal receptor positive agreed to hormonal therapy. Compliance to recommendation of hormonal therapy was 61.7%.

It is well reported in the literature that EPC has a good prognosis with 10‐year survival of close to 100%.^15^ The largest reported study of 917 cases of patients with EPC in California reported similar relative cumulative survival rate in EPC alone group and EPC with invasive cancer group, 96.8% and 94.4% respectively.^21^ Usually, in the absence of concomitant DCIS or IDC, EPC has favourable prognosis with only small numbers of lymph node metastasis and no disease related deaths.^18^ Our study supports the results, with overall survival of 85% at 5 years follow up and median overall survival of 58.5 (1–241) months. Morever, the cause of deaths for those patients are not breast cancer related—mainly ischaemic heart disease and hepatobiliary cancer. Disease free survival at 5 years was 100% for EPCs with DCIS and EPCs with IDC, while pure EPC reported a disease free survival of 96%.

We recognise the limitation of our study, which includes the small number of patients, given the rarity of this disease, and the retrospective nature of the analysis and variety in biopsy techniques used. Despite these limitations, our study demonstrated that the overall outcomes after treatment of EPC are excellent. Given the good prognosis of patients with EPC, a much longer follow up duration is would be required to detect any significant differences, if any, in the overall survival between the different subgroups of EPCs. A prospective randomised trial should be suggested to further characterise the disease and treatment outcomes.

In conclusion, EPC is a rare tumour mainly affecting post‐menopausal woman with excellent prognosis, with no significant differences in overall survival between all three groups—pure EPC, EPC with DCIS and EPC with IDC. The presence of a solid component within a cystic lesion of the breast should raise the clinician's suspicion of malignancy, and due care has to be taken when doing a core biopsy, to ensure samples are obtained from the solid component of these lesions. Current data further support treatment of pure EPC in a similar manner to DCIS. Although lymph node involvement is extremely rare in patients with pure EPC, sentinel lymph node biopsy should be considered in patients with EPC associated with IDC or in patients undergoing mastectomy. We hope the experience of our institution will contribute the available data on this rare tumour, and supplement to a better understanding of this unique subgroup.

## AUTHOR CONTRIBUTIONS

**Hiang Jin Tan:** Conceptualization (equal); data curation (equal); formal analysis (equal); methodology (equal); project administration (equal); writing – original draft (equal); writing – review and editing (equal). **Puay Hoon Tan:** Formal analysis (equal); resources (equal); supervision (equal). **Lester Chee Hao Leong:** Resources (equal); supervision (equal); validation (equal); writing – review and editing (equal). **Veronique Kiak Mien Tan:** Supervision (equal); validation (equal); visualization (equal). **Benita Kiat Tee Tan:** Resources (equal); supervision (equal); validation (equal); writing – review and editing (equal). **Sue Zann Lim:** Resources (equal); supervision (equal); validation (equal); writing – review and editing (equal). **Madhukumar Preetha:** Resources (equal); software (equal); supervision (equal); validation (equal); writing – review and editing (equal). **Chow Yin Wong:** Software (equal); supervision (equal); visualization (equal). **Wei Sean Yong:** Resources (equal); software (equal); supervision (equal); validation (equal). **Yirong Sim:** Conceptualization (equal); data curation (equal); investigation (equal); methodology (equal); software (equal); supervision (equal); validation (equal); visualization (equal); writing – review and editing (equal).

## FUNDING INFORMATION

There are no sources of funding with this manuscript.

## CONFLICT OF INTEREST STATEMENT

The authors declare that there is no conflict of interest.

## Data Availability

n/a.
